# Carcinome épidermoïde de la vésicule biliaire: à propos d'un cas

**DOI:** 10.11604/pamj.2015.22.382.8350

**Published:** 2015-12-29

**Authors:** Mohamed Hedfi, Taieb Jomni, Cherif Abdelhedi, Karim Sassi, Adnene Chouchene

**Affiliations:** 1 Hôpital des FSI, La Marsa, Rue Fadhel, Ben Achour 2070, Tunisie

**Keywords:** Carcinome, vésicule biliaire, cholestase anictérique, cholécystectomie, Carcinoma, gall blader, anicteric cholestasis, cholecystectomy

## Abstract

Patient âgé de 59 ans ayant consulté pour un syndrome douloureux et fébrile de l'hypochondre droit évoluant depuis quatre jours. La palpation trouvait une défense et la biologie montrait un syndrome inflammatoire biologique associé à une cholestase anictérique. A l’échographie la vésicule biliaire était distendue, multi lithiasique, à paroi épaissie, associée à un aspect hypoéchogène du segment IV du foie et une thrombose de la branche portale gauche. L'exploration per opératoire, montrait un pyocholécyste, une vésicule biliaire enchâssée dans le foie avec une importante pédiculite et un segment IV induré sans abcès hépatique. Une cholécystectomie avec drainage étaient réalisées. L'examen anatomopathologique était en faveur d'un carcinome épidermoïde bien différencié occupant toute la vésicule biliaire et infiltrant toute la paroi en dépassant la séreuse. Cette tumeur était classée stade pT3 selon la classification TNM et la limite d'exérèse proximale était tumorale. Malgré une chimiothérapie palliative à base de Gemzar- Cisplatine l’évolution était rapidement défavorable.

## Introduction

Le carcinome épidermoïde vésiculaire est une tumeur très rare, représentant moins de 2% des tumeurs malignes de la vésicule [1]. Il est caractérisé par une progression rapide vers un envahissement locorégional et des métastases hépatiques. Son caractère invasif, associé à un diagnostic souvent tardif expliquent son très mauvais pronostic. Nous rapportons une nouvelle observation d'un carcinome épidermoïde primitif de la vésicule biliaire en discutant les particularités cliniques, histologiques et thérapeutiques de cette entité.

## Patient et observation

**Figure 1:**
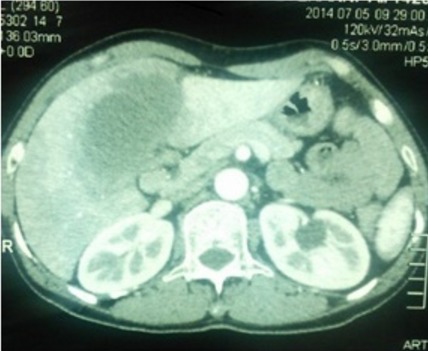
Vésicule biliaire distendue, entourée de plages hypodenses du segment IV en rapport avec des foyers de suppuration

**Figure 2:**
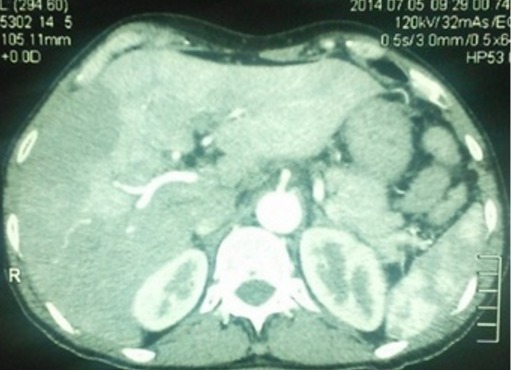
Thrombose portale gauche partielle avec troubles perfusionnels hépatiques

**Figure 3:**
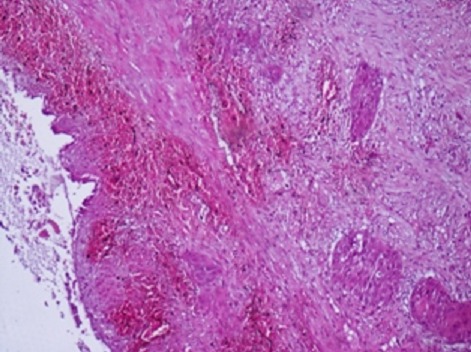
Prolifération carcinomateuse infiltrant à différenciation épidermoïde agencée en amas. Le revêtement de type biliaire de surface est d'aspect régénératif

Il s'agissait d'un patient âgé de 59 ans, tabagique à 40PA, aux antécédents d'ulcère gastroduodénal traité médicalement il y a 20 ans. Il a consulté pour syndrome douloureux et fébrile de l'hypochondre droit évoluant depuis quatre jours sans trouble du transit. A l'examen, il était subfébrile à 38°C, anictérique, et la palpation trouvait une défense de l'hypochondre droit. A la biologie, la CRP était à 136mg/l, les γGT et PAL étaient légèrement élevés à 1.5 fois la normale. Les transaminases, les globules blancs, l'amylasémie ainsi que la lipasémie étaient normales. Une échographie abdominale montrait une vésicule biliaire (VB) distendue multi lithiasique à paroi épaissie, siège d'un sludge et une lame d’épanchement sous hépatique autour du lit vésiculaire sans dilatation des voies biliaires intra ni extra hépatiques. Le segment IV était hypoéchogène avec une thrombose de la branche portale gauche et des ganglions au niveau du hile hépatique. La TDM abdominale objectivait une VB distendue entourée de collections hépatiques perivésiculaires confluentes associées à une thrombose porte gauche partielle (Figure 1, Figure 2). Le patient a été opéré par voie sous costale droite. L'exploration per opératoire, montrait un pyocholécyste, une VB enchâssée dans le foie avec une importante pédiculite et un segment IV induré sans abcès hépatique. Une cholécystectomie avec drainage étaient réalisées avec des suites immédiates simples. L'examen anatomopathologique était en faveur d'un carcinome épidermoîde bien différencié occupant toute la VB et infiltrant toute la paroi en dépassant la séreuse (Figure 3). La limite d'exérèse chirurgicale proximale était tumorale, sans embols vasculaires ni engainement peri nerveux. Cette tumeur était classée stade p T3 selon la classification TNM. Une TDM thoraco-abdominale faite un mois après l'intervention montrait un envahissement des segments II, IV, VI et VII avec importante infiltration de la graisse autour et persistance de la thrombose porte gauche. Une chimiothérapie palliative à base de Gemzar- Cisplatine était entamée puis arrêtée après 5 cures suite une altération rapide de l’état général et la survenue d'une hémorragie digestive haute.

## Discussion

Le cancer de la vésicule biliaire ne représente que 3 à 4% des cancers digestifs. Il reste de mauvais pronostic et peu étudié du fait de sa rareté [1]. Encore plus rare, le carcinome épidermoîde est une entité histologique qui ne représente que moins de 2% des cancers de la VB [2, 3]. Son épidémiologie est similaire aux adénocarcinomes vésiculaires avec un sex ratio de 3/1 et un âge moyen de survenue entre 40 et 60 ans [4]. Son évolution est caractérisée par sa capacité de prolifération deux fois plus rapide que celle de la composante glandulaire [5]. Cette dernière tend à envahir les ganglions lymphatiques, les structures vasculaires et les voies biliaires, tandis que la composante épidermoïde possède une agressivité surtout locale avec envahissement fréquent des structures adjacentes comme le foie, le tractus biliaire, le duodénum ou l'angle colique droit, ce qui explique les limites de sa résécabilité et le moins bon pronostic par rapport à l'adénocarcinome. En effet, le devenir de ces patients serait uniquement amélioré par une résection sans résidu tumoral (R0), quand celle-ci est possible [6]. Comme pour les autres cancers vésiculaires, le rôle éthiopathogénique d'une lithiase vésiculaire sous-jacente présente dans 60 à 90% des cas est évoqué. Dans une étude menée par Cariati et al [7], colligeant 75 cas de cancers vésiculaires de la période allant de 1986 à 2012, l'association à des macrolithiases a été observée dans 88,88% des cas pour les carcinomes épidermoides contre 68.2% des cas pour les adénocarcinomes. La carcinogenèse des lithiases vésiculaires pourrait résulter de traumatismes itératifs et de l'irritation chronique. Cependant, des dérivés carcinogènes des acides biliaires peuvent également jouer un rôle [8]. L'histogenèse de ces tumeurs est toujours obscure mais il est possible que ces tumeurs dérivent d'une lignée cellulaire exprimant deux phénotypes. Des cellules métaplasiques squameuses pourraient ainsi se développer en réponse à une irritation d'un adénocarcinome pré existant réalisant une métaplasie d'un adénocarcinome en cellules épithéliales [9, 10]. L'association de cellules squameuses métaplasiques et adénomateuses dans certaines études sont en faveur de cette hypothèse [3, 10]. Les signes cliniques sont peu spécifiques et ressemblent aux autres tumeurs vésiculaires qui ont toutes la particularité de se manifester tardivement à un stade évolué de la maladie [4]. Takeyoshi et al ont rapporté un cas clinique ou la découverte était à un stade infra clinique sur une pièce de cholécystectomie pour cholécystite aigue [9]. Le traitement curatif des carcinomes épidermoîdes ne diffère pas des autres carcinomes et dépend de l'extension locorégionale. Il demeure basé sur la résection chirurgicale, étendue sur le lit vésiculaire. Malgré la faible extension lymphatique, qui est une caractéristique du carcinome épidermoide, un curage ganglionnaire est recommandé [10]. Seule la résection R0 peut améliorer le pronostic de ces carcinomes epidermoides. Comme pour les adénocarcinomes vésiculaires, la réalisation d'une cholécystectomie chez les patients à haut risque (calcul de plus de 3cm, polype >1cm, reflux pancreatico-biliaire, vésicule en porcelaine, adénomyomateuse segmentaire, cholecystite xanthogranulomateuse) reste encore le moyen de prévention le plus performant. La thérapie combinant la gemcitabine et la cisplatine prescrite pour notre patient est considérée comme le traitement palliatif standard indiqué pour les cancers irrésécables [10]. L'utilisation des agents de ciblage moléculaire moins toxiques pourraient être aussi faire partie du potentiel thérapeutique de ces cancers dans le futur (Figure 3).

## Conclusion

Les carcinomes épidermoïdes de la vésicule biliaire sont des tumeurs rares. Pour améliorer le pronostic qui reste malheureusement sombre, il est nécessaire de réaliser des études multicentriques colligeant plusieurs cas afin de mieux dégager les caractéristiques cliniques, oncogénétiques ainsi que d’éventuelles implications thérapeutiques.
